# Piezo1 opposes age‐associated cortical bone loss

**DOI:** 10.1111/acel.13846

**Published:** 2023-05-05

**Authors:** Xuehua Li, Connie Zhang, Hayden H. Bowman, Jeffrey B. Stambough, Benjamin M. Stronach, Simon C. Mears, Lowry C. Barnes, Elena Ambrogini, Jinhu Xiong

**Affiliations:** ^1^ Department of Orthopaedic Surgery University of Arkansas for Medical Sciences Little Rock Arkansas USA; ^2^ Center for Musculoskeletal Disease Research University of Arkansas for Medical Sciences Little Rock Arkansas USA; ^3^ Division of Endocrinology University of Arkansas for Medical Sciences Little Rock Arkansas USA

**Keywords:** age‐associated bone loss, endocortical bone resorption, mechanical stimulation, Piezo1

## Abstract

As we age, our bones undergo a process of loss, often accompanied by muscle weakness and reduced physical activity. This is exacerbated by decreased responsiveness to mechanical stimulation in aged skeleton, leading to the hypothesis that decreased mechanical stimulation plays an important role in age‐related bone loss. Piezo1, a mechanosensitive ion channel, is critical for bone homeostasis and mechanotransduction. Here, we observed a decrease in *Piezo1* expression with age in both murine and human cortical bone. Furthermore, loss of *Piezo1* in osteoblasts and osteocytes resulted in an increase in age‐associated cortical bone loss compared to control mice. The loss of cortical bone was due to an expansion of the endosteal perimeter resulting from increased endocortical resorption. In addition, expression of *Tnfrsf11b*, encoding anti‐osteoclastogenic protein OPG, decreases with *Piezo1* in vitro and in vivo in bone cells, suggesting that Piezo1 suppresses osteoclast formation by promoting *Tnfrsf11b* expression. Our results highlight the importance of Piezo1‐mediated mechanical signaling in protecting against age‐associated cortical bone loss by inhibiting bone resorption in mice.

## Graphical abstract

Age‐related bone loss is associated with decreased expression of Piezo1, a mechanosensitive ion channel, and osteoprotegerin (OPG), an osteoclastogenesis inhibitory factor. Loss of Piezo1 signaling in osteoblasts and osteocytes exacerbates cortical bone loss in old mice by enhancing osteoclast formation in endocortical bone surface. Our results indicate the importance of Piezo1‐mediated signaling in protecting against age‐associated cortical bone loss.

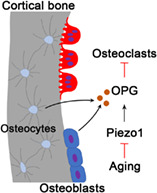

**Abbreviations**
Acp5: acid phosphatase 5
BAPTA: 1,2‐bis(o‐aminophenoxy) ethane‐N,N,N′,N′‐tetra acetic acid
CaM: calmodulin
Cre: Cre recombinase
CtsK: cathepsin K
Dmp1: dentin matrix acidic phosphoprotein 1
ERK: extracellular signal‐regulated kinase
eNOS: endothelial nitric oxide synthase
m‐CSF: macrophage colony‐stimulating factor
MEK: mitogen‐activated protein kinase kinase
MLO‐Y4: murine long bone osteocyte Y4
mTOR: mammalian target of rapamycin
RANKL: receptor activator of nuclear factor kappa‐Β ligand
Tnfrsf11b: tumour necrosis factor receptor superfamily member 11b
TRAP: tartrate‐resistant acid phosphatase

## 1 INTRODUCTION

Advanced age causes bone loss in both cancellous and cortical compartments (Almeida et al., 2007; Piemontese et al., 2017; Riggs et al., 2008). The cancellous bone loss with age is associated with decreased bone formation and a low rate of bone remodeling (Almeida et al., 2007). In contrast, age‐associated cortical bone loss is mainly due to increased osteoclast number and bone resorption at the endocortical surface (Piemontese et al., 2017). Our previous studies showed that *Tnfsf11* encoding RANKL, a cytokine that is essential for osteoclast formation, increases with age in cortical bone (Piemontese et al., 2017). In addition, OPG, a decoy receptor for RANKL, decreases with age (Piemontese et al., 2017). More importantly, blockade of osteoclast formation by deletion of *Tnfsf11* in osteocytes prevents the increase in osteoclast number and cortical bone loss with age in mice (Kim et al., 2020). These studies demonstrate the importance of increased osteoclast formation in age‐associated cortical bone loss.

In humans and rodents, the loss of bone mass caused by aging is often associated with a decline in muscle mass and physical activity (Curtis et al., 2015; Hamrick et al., 2006). These declines lead to reduced mechanical loading on bone. Mechanical stimulation such as that derived from physical activity promotes bone formation by increasing osteoblast number and activity (Bailey & Brooke‐Wavell, 2010; Robling & Turner, 2009; Turner et al., 1998). On the contrary, loss of mechanical stimulation causes bone loss by promoting osteoclast formation and bone resorption (Xiong et al., 2011). Therefore, both osteoblasts and osteoclasts are regulated by mechanical stimulation. In addition, the anabolic response to mechanical loading in bone decreases with age indicating a reduced ability to sense and/or transduce mechanical stimulation in old bone (Holguin et al., 2014; Karinkanta et al., 2007). Taken together, these findings suggest that decreased mechanical stimulation contributes to age‐associated bone loss.

Piezo1, a mechanosensitive ion channel, is capable of sensing various mechanical stimulation including membrane stretch, matrix rigidity, and fluid shear stress and plays an important role in mechanotransduction in many organs (Coste et al., 2010; Gudipaty et al., 2017; Li et al., 2014, 2021; Miyamoto et al., 2014). We and others have deleted the *Piezo1* gene in osteoblast lineage cells and demonstrated that Piezo1 plays a critical role in bone homeostasis (Hendrickx et al., 2021; Li et al., 2019; Sun et al., 2019; Zhou et al., 2020). In our studies, we found that cancellous bone mass was decreased in young adult mice lacking *Piezo1* in *Dmp1‐Cre* targeted cells. We also observed decreased cortical thickness and periosteal circumference in these mice. More importantly, the skeletal response to mechanical loading was blunted in the conditional knockout mice (Hendrickx et al., 2021; Li et al., 2019). These results demonstrate that *Piezo1* expression in *Dmp1‐Cre* targeted cells is essential for bone homeostasis and mediates the skeletal response to mechanical stimulation. In present study, we determined whether *Piezo1* expression in osteoblast lineage cells plays an important role in age‐associated bone loss. We found that mice lacking *Piezo1* in osteoblast lineage cells lose more cortical bone with age than control mice and that this is associated with a more profound increase in osteoclast number at the endocortical surface.

## 2 RESULTS

### 2.1 *Piezo1* expression decreases with age

**FIGURE 1:**
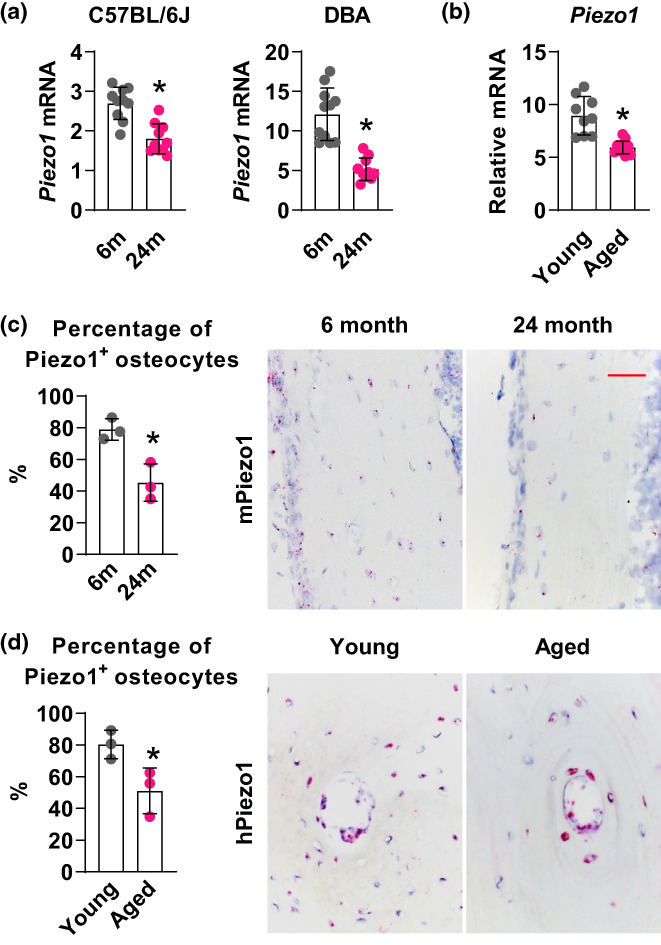
*Piezo1* expression decreases with age. (a) mRNA levels of *Piezo1* in 6‐ and 24‐month‐old C57BL/6J and DBA mice (*n* = 9–11, here and throughout, values are mean ± SD). (b) qPCR of *Piezo1* mRNA in human cortical bone isolated from human femoral neck removed during hip replacement surgeries from young (*n* = 9 with mean age of 47.1 ± 7.01 years old) and aged patients (*n* = 13 with mean age of 73.8 ± 3.2 years old). (c) Quantification and representative images of *Piezo1* mRNA in situ hybridization using RNAscope on femoral cortical bone of 6‐ and 24‐month‐old C57BL/6J mice. Red dots indicate positive *Piezo1* signals. Scale bar, 100 μm. (d) Quantification and representative images of *Piezo1* mRNA in situ hybridization on human cortical bone of femoral neck from young and old individuals. Red dots indicate positive *Piezo1* signals. *p < 0.05 using Student's *t* test.

To determine whether *Piezo1* expression changes with advanced age, we compared *Piezo1* expression in tibia cortical bone of 6‐month‐old versus 24‐month‐old mice and found that *Piezo1* expression is significantly reduced in cortical bone of 24‐month‐old mice (Figure 1a). This decrease was observed in both the C57BL/6J and DBA mouse strains (Figure 1a). In addition to mice, the expression of *Piezo1* was also compared between young and old human subjects in the cortical bone of femoral neck. The results showed a similar decrease in *Piezo1* expression in the elderly human subjects (73.8 ± 3.2 years old) compared to the young ones (47.1 ± 7.01 years old) (Figure 1b). To further validate the findings, RNAscope, an RNA in situ hybridization technique, was performed on the mouse and human bone samples. The results indicated decreased *Piezo1* mRNA in cortical bone from old murine femur and human femoral neck (Figure 1c,d), which is consistent with the gene expression analysis. Overall, the results provide compelling evidence of a correlation between aging and decreased *Piezo1* expression in bone. The findings suggest that the reduction in *Piezo1* expression may play a role in the age‐related changes in bone.

### 2.2 Deletion of *Piezo1* in osteoblasts and osteocytes exacerbates cortical bone loss with age

To determine whether *Piezo1* expression in osteoblast lineage cells plays a role in age‐associated bone loss, we generated *Piezo1* conditional knockout mice in which the *Piezo1* gene is deleted from *Dmp1‐Cre* transgene targeted cells, hereafter referred to as Dmp1‐Cre; Piezo1^f/f^ mice. Littermates homozygous for the conditional allele but lacking the *Dmp1‐Cre* transgene were used as controls. These mice were aged to 6 and 24 months of age, and the bones of 24‐month‐old mice were then analyzed and compared with bones from 6‐month‐old mice by micro‐CT analysis.

**FIGURE 2:**
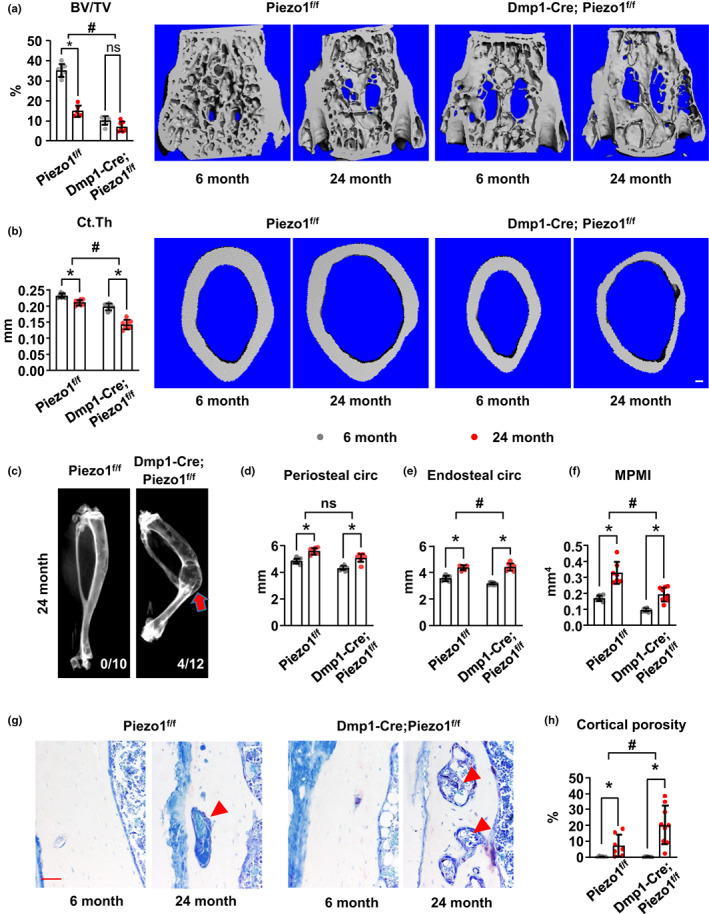
Deletion of *Piezo1* in osteoblasts and osteocytes exacerbates the cortical bone loss with age. (a) Cancellous bone volume per tissue volume (BV/TV) and the representative μCT images measured in the L4 vertebra of 6‐ and 24‐month‐old female Piezo1^f/f^ (*n* = 8, 10) and Dmp1‐Cre; Piezo1^f/f^ (*n* = 8, 12) mice. (b) Cortical thickness and the representative μCT images (scale bar, 1 mm) measured in the femoral diaphysis of 6‐ and 24‐month‐old female Piezo1^f/f^ (*n* = 8, 10) and Dmp1‐Cre; Piezo1^f/f^ (*n* = 8, 12) mice. (c) X‐ray images of tibia from 24‐month‐old Piezo1^f/f^ and Dmp1‐Cre; Piezo1^f/f^ littermate. Fractions represent 0 and 4 mice displayed tibia facture out of 10 and 12 mice, respectively. Arrow indicates the location of fracture. (d–f) Periosteal circumference (d), endocortical circumference (e), and moment of inertia (f) analysis of the femoral diaphysis by micro‐CT from 6‐ and 24‐month‐old female Piezo1^f/f^ (*n* = 8, 10) and Dmp1‐Cre; Piezo1^f/f^ (*n* = 8, 12) mice. (g) Representative histological longitudinal section images of cortical bone showing cortical porosity in 6‐ and 24‐month‐old female Piezo1^f/f^ and Dmp1‐Cre; Piezo1^f/f^ mice. Red arrow heads indicate pores in cortical bone. Scale bar, 50 μm. (h) Cortical porosity measured by μCT in femur from 6‐ and 24‐month‐old female Piezo1^f/f^ (*n* = 8, 10) and Dmp1‐Cre; Piezo1^f/f^ (*n* = 8, 12) mice. *p < 0.05 with the comparisons indicated by the brackets using 2‐way ANOVA. #, *p* < 0.05 for interaction using 2‐way ANOVA. ns, nonsignificant.

As expected, control mice exhibited age‐associated cancellous bone loss at 24 months of age compared to 6‐month‐old mice (Figure 2a; Figure S1a). In contrast, conditional knockout mice, which had lower cancellous bone mass than controls at young age, did not further lose cancellous bone with age (Figure 2a; Figure S1a). Analysis of femoral cortical bone revealed that control mice had lower cortical thickness at 24 versus 6 months of age (Figure 2b). Interestingly, old Dmp1‐Cre; Piezo1^f/f^ mice displayed a more profound decrease in cortical thickness with age compared to control mice (Figure 2b). Consistent with this, 24‐month‐old Dmp1‐Cre; Piezo1^f/f^ mice displayed spontaneous tibial fracture (4 out of 12), whereas no fracture was observed in either young mice or old controls (Figure 2c). These results demonstrate that even starting with lower cortical thickness, mice lacking *Piezo1* in osteoblasts and osteocytes lose more cortical bone with age.

To identify the sites of the cortical bone loss, we measured periosteal and endosteal circumference in the midshaft of femur. Micro‐CT analysis revealed that the periosteal circumference increased with age in control mice (Figure 2d). Despite the lower periosteal circumference established during growth, adult Dmp1‐Cre; Piezo1^f/f^ mice displayed a comparable increase in periosteal circumference with age as control mice (Figure 2d). The increase of total cross‐sectional area with age in femoral midshaft was also comparable between two genotypes (Figure S1b). Endosteal circumference was increased in both control and Dmp1‐Cre; Piezo1^f/f^ mice at 24 months of age (Figure 2e). However, this increase was greater in conditional knockout mice than that in control mice (Figure 2e). In line with this, the increase of medullary area with age was more profound in conditional knockout mice, whereas the increase of cortical area was blunted in these mice (Figure S1b). Consistently, the increase in polar moment of inertia with age measured in femoral midshaft was blunted in the Dmp1‐Cre; Piezo1^f/f^ mice (Figure 2f). Like cortical thickness, Dmp1‐Cre; Piezo1^f/f^ mice developed more cortical porosity with age than control mice as shown in histological sections and by micro‐CT analysis (Figure 2g,h). Taken together, these results demonstrate that *Piezo1* deletion in Dmp1‐Cre targeted cells increased endocortical expansion and cortical porosity in old mice, suggesting that the greater loss of cortical bone with age in conditional knockout mice is due to increased endocortical bone resorption.

### 2.3 Deletion of *Piezo1* in osteoblasts and osteocytes increases osteoclast formation at endocortical surface

**FIGURE 3:**
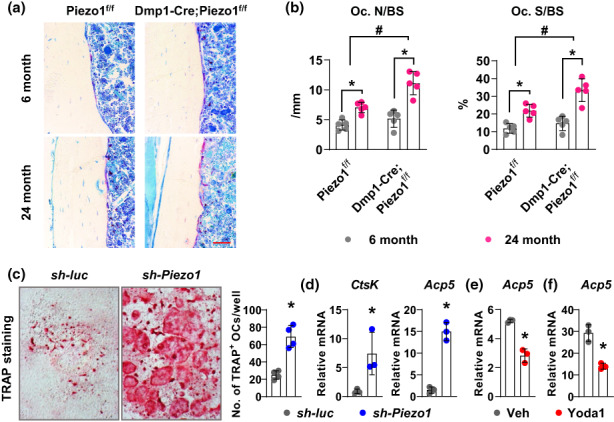
Deletion of *Piezo1* in osteoblasts and osteocytes increases osteoclast formation at endocortical surface. (a) Representative images of TRAP staining at the endocortical surface of 6‐ and 24‐month‐old female Piezo1^f/f^ and Dmp1‐Cre; Piezo1^f/f^ mice. Scale bar, 50 μm. (b) Osteoclast number (N.Oc/B.Pm, left) and osteoclast surface (Oc.S/BS, right) measured at femoral endocortical surface of 6‐ and 24‐month‐old female Piezo1^f/f^ (*n* = 5, 5) and Dmp1‐Cre; Piezo1^f/f^ (*n* = 5, 5) mice. *p < 0.05 with the comparisons indicated by the brackets using 2‐way ANOVA. #, *p* < 0.05 for interaction using 2‐way ANOVA. (c) Representative images of TRAP staining and quantification of TRAP^+^ osteoclasts in cocultures of bone marrow derived macrophages with MLO‐Y4 cells with or without *Piezo1* knocked down. (d) mRNA of osteoclastic genes *Ctsk* and *Acp5* in cocultures of bone marrow derived macrophages with MLO‐Y4 cells with or without *Piezo1* knocked down. (e) mRNA of *Acp5* in cocultures of bone marrow derived macrophages with MLO‐Y4 cells in the presence of Piezo1 agonist Yoda1. (f) *Acp5* expression in femoral cortical bone cultured ex vivo in the presence of Piezo1 agonist Yoda1. *p < 0.05 using Student's *t* test.

To understand the cellular basis of the skeletal phenotype, we measured osteoclast number and surface at the femoral endocortical surface. TRAP staining showed that osteoclast number and surface in endosteum were dramatically increased in aged mice than that in young mice in both genotypes (Figure 3a,b). Moreover, this increase was significantly elevated in conditional knockout mice (Figure 3a,b). To determine whether loss of *Piezo1* in osteocytes enhances their ability to support osteoclast formation, we knocked down *Piezo1* in MLO‐Y4 cells, an osteocyte‐like cell line, using shRNA and co‐cultured these cells with bone marrow derived macrophages in the presence of M‐CSF. After 7 days of coculture, cells were fixed for TRAP staining or harvested for gene expression. TRAP staining showed that *Piezo1* knock‐down cells have greater ability to support osteoclast formation than control cells (Figure 3c). Consistent with the staining, gene expression analysis showed increased osteoclast marker genes including *CtsK* and *Acp5* in *Piezo1* knockdown cultures (Figure 3d). In contrast, activation of Piezo1 using its small molecular agonist Yoda1 inhibits the ability of osteocytes to support osteoclast formation indicated by decreased *Acp5* expression after 7 days of coculture (Figure 3e). In addition, we cultured femoral cortical bone ex vivo and treated the bone samples with Yoda1 for 3 days. We found that Yoda1 decreased *Acp5* expression in ex vivo organ culture of femoral cortical bone (Figure 3f). Taken together, these results demonstrate that loss of *Piezo1* promotes osteoclast formation.

### 2.4 Deletion of *Piezo1* in osteoblasts and osteocytes suppresses *Tnfrsf11b* expression

**FIGURE 4:**
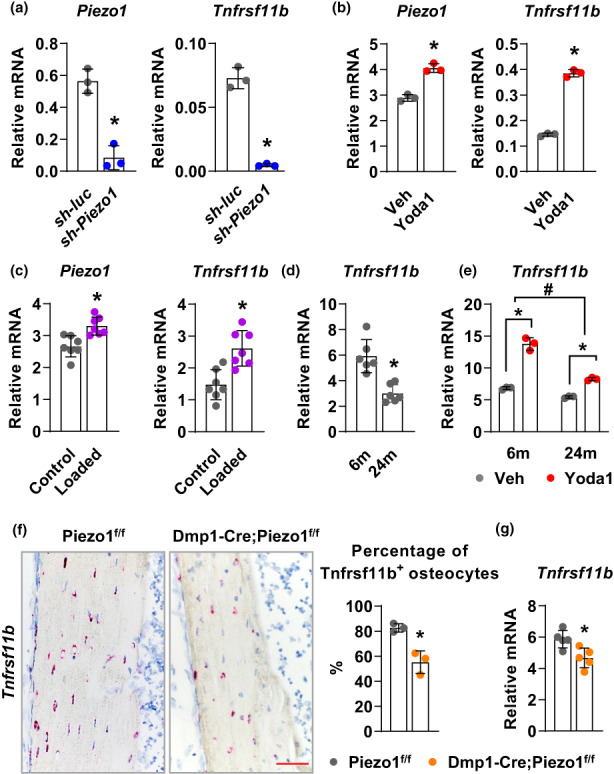
Deletion of *Piezo1* in osteoblasts and osteocytes suppresses *Tnfrsf11b* expression. (a) qPCR of *Piezo1* and *Tnfrsf11b* mRNA in control or *Piezo1* knock‐down MLO‐Y4 cells. *p < 0.05 using Student's *t* test. (b) Relative mRNA levels of *Piezo1* and *Tnfrsf11b* in MLO‐Y4 cells treated with 10 μM Yoda1 for 2 h. *p < 0.05 using Student's *t* test. (c) *Piezo1* and *Tnfrsf11b* mRNA levels measured in tibia of 3‐month‐old female C57BL/6J (*n* = 7) mice loaded with one bout of compressive loading. Mice were harvested 4 h after loading. *p < 0.05 using Student's *t* test. (d) *Tnfrsf11b* mRNA levels in femoral cortical bone of 6‐ and 24‐month‐old female C57BL/6J mice (*n* = 6). *p < 0.05 using Student's *t* test. (e) *Tnfrsf11b* mRNA levels in Veh or Yoda1 treated bone marrow stromal cells isolated from 6‐ and 24‐month‐old female C57BL/6J mice. *p < 0.05 with the comparisons indicated by the brackets using 2‐way ANOVA. #, *p* < 0.05 for interaction using 2‐way ANOVA. (f) Representative images and quantification of *Tnfrsf11b* mRNA in situ hybridization using RNAscope on femoral cortical bone of 3‐month‐old female Piezo1^f/f^ and Dmp1‐Cre; Piezo1^f/f^ mice (*n* = 3). Red dots indicate positive *Tnfrsf11b* signals. Scale bar, 100 μm. (g) *Tnfrsf11b* mRNA levels in osteocyte‐enriched femoral cortical bone of 4‐month‐old male Piezo1^f/f^ and Dmp1‐Cre; Piezo1^f/f^ mice (*n* = 5). *p < 0.05 using Student's *t* test.

To further understand the mechanisms by which Piezo1 regulates osteoclast formation, we analyzed the expression levels of genes known to play a crucial role in osteoclastogenesis, including *Tnfsf11* and *Tnfrsf11b* in *Piezo1* knock‐down MLO‐Y4 cells. Our results revealed that *Tnfrsf11b* expression was dramatically decreased in *Piezo1* knock‐down cells (Figure 4a). Conversely, activation of Piezo1 by Yoda1 resulted in a marked increase in *Tnfrsf11b* expression in MLO‐Y4 cells (Figure 4b). Consistently, mechanical stimulation promoted *Piezo1* expression and *Tnfrsf11b* expression in cortical bone in mice (Figure 4c). These data suggest that Piezo1 controls osteoclast formation by stimulating *Tnfrsf11b* expression in bone cells. In addition, *Tnfrsf11b* expression decreases with age, similar to *Piezo1* expression in old mice (Figure 4d). Importantly, the increase in *Tnfrsf11b* induced by Yoda1 was significantly blunted in bone cells isolated from old mice compared to those from young mice (Figure 4e). As a result, loss of Piezo1 could further decrease *Tnfrsf11b* expression in old mice. To determine *Tnfrsf11b* expression in *Piezo1* knockout mice, we performed RNAscope to measure *Tnfrsf11b* expression in cortical bone. In control mice, RNAscope showed that about 82% of osteocytes express *Tnfrsf11b* mRNA (Figure 4f). However, in knockout mice, the percentage of osteocytes that express *Tnfrsf11b* decreased to 56% (Figure 4f). Consistently, gene expression analysis of osteocyte‐enriched cortical bone also showed a reduction in *Tnfrsf11b* expression in *Piezo1* conditional knockout mice (Figure 4g). Therefore, our results indicate that Piezo1 controls osteoclast formation via upregulation of *Tnfrsf11b* expression.

### 2.5 Piezo1 controls *Tnfrsf11b* expression via Ca^2+^/CaM/mTOR signaling pathway

**FIGURE 5:**
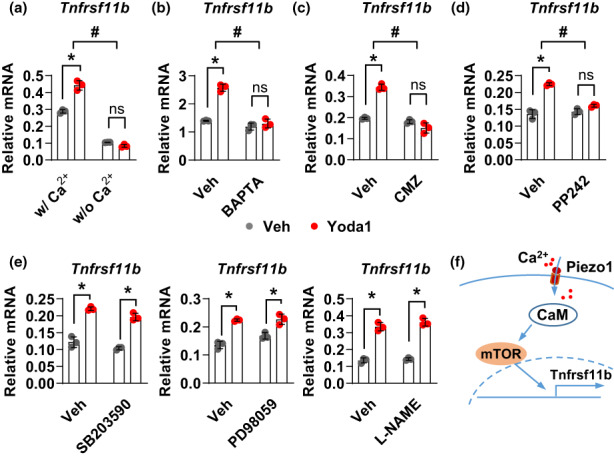
Piezo1 controls *Tnfrsf11b* expression via Ca^2+^/CaM/mTOR signaling pathway. (a) qPCR of *Tnfrsf11b* mRNA in MLO‐Y4 cells treated with Veh or 10 μM Yoda1 for 2 h. Cells were cultured in αMEM either with or without calcium. (b) Relative mRNA levels of *Tnfrsf11b* in MLO‐Y4 cells treated with Veh or 10 μM Yoda1 for 2 h in the presence of 5 μM intracellular calcium chelator BAPTA. (c) Relative mRNA levels of *Tnfrsf11b* in MLO‐Y4 cells treated with 10 μM Yoda1 for 2 h in the presence of 1.5 μM calmidazolium (CMZ), a membrane‐permeable CaM antagonist. (d) Relative mRNA levels of *Tnfrsf11b* in MLO‐Y4 cells treated with Veh or 10 μM Yoda1 for 2 h in the presence of 2.5 μM mTOR inhibitor PP242. (e) Relative mRNA levels of *Tnfrsf11b* in MLO‐Y4 cells treated with Veh or 10 μM Yoda1 for 2 h in the presence of 10 μM SB203590 (p38/MAPK inhibitor), 50 μM PD98059 (MEK/ERK inhibitor), and 200 μM L‐NAME (eNOS inhibitor). (f) Schematic illustration of the pathway by which Piezo1 controls *Tnfrsf11b* expression. *n* = 3 per group. Each experiment was repeated at least two times. *p < 0.05 with the comparisons indicated by the brackets using 2‐way ANOVA. #, *p* < 0.05 for interaction using 2‐way ANOVA.

To further investigate the mechanisms by which Piezo1 regulates *Tnfrsf11b* expression, we used MLO‐Y4 cells as a model. We first examined whether calcium influx is required for Yoda1 induced *Tnfrsf11b* expression since Piezo1 is a calcium permissive ion channel and Yoda1 induces calcium influx in MLO‐Y4 cells (Li et al., 2019). We cultured MLO‐Y4 cells in calcium free medium and treated these cells with Yoda1 for 2 h. We found that Yoda1 induced *Tnfrsf11b* expression was completely blunted in the absence of extracellular calcium (Figure 5a). We then determined whether intracellular calcium is required for Yoda1 induced *Tnfrsf11b* expression. We treated MLO‐Y4 cells with BAPTA, an intracellular calcium chelator, and found that Yoda1 induced *Tnfrsf11b* expression was blocked without free intracellular calcium (Figure 5b). We next examined the effect of calmodulin (CaM), a calcium‐binding protein that mediates intracellular calcium signaling, on the increase of *Tnfrsf11b* expression induced by Yoda1. We treated cells with Yoda1 in the presence of calmidazolium (CMZ), a membrane‐permeable CaM antagonist. CMZ diminished the increase of *Tnfrsf11b* expression (Figure 5c). Thus, Ca^2+^ and CaM are required for the increase of *Tnfrsf11b* expression in response to Piezo1 activation. Ca^2+^/CaM has been shown to regulate various signaling pathways including p38/MAPK, MEK/ERK, eNOS, and mTOR signaling (Agell et al., 2002; Fleming et al., 2001; Gulati et al., 2008; Krapivinsky et al., 2004). In addition, Piezo1 has been shown to regulate p38, eNOS, and mTOR signaling in endothelial cells (Blythe et al., 2019; Wang et al., 2016; Wang et al., 2021). Therefore, we utilized inhibitors of each these pathways to determine whether they are required for *Tnfrsf11b* expression induced by Piezo1. Our results revealed that mTOR inhibitor PP242 blocked Yoda1 induced *Tnfrsf11b* expression (Figure 5d). Other pathway inhibitors including SB203590 (p38/MAPK inhibitor), PD98059 (MEK/ERK inhibitor), and L‐NAME (eNOS inhibitor) did not affect the increase of *Tnfrsf11b* expression induced by Piezo1 activation (Figure 5e). These results indicate that Piezo1 controls *Tnfrsf11b* expression via the Ca^2+^/CaM/mTOR signaling pathway (Figure 5f).

## 3 DISCUSSION

Loss of mechanical stimulation has been implicated in age‐associated bone loss (Javaheri & Pitsillides, 2019). In the current study, we investigated the role of Piezo1, a mechanosensor, in age‐associated bone loss. Our studies showed that the increased endocortical bone resorption that occurs with old age is associated with a decline of Piezo1 expression in bone. Moreover, our results indicate that Piezo1 plays an important role in preventing age‐associated increase in endocortical bone resorption by maintaining OPG expression. Therefore, we provide evidence that Piezo1 signaling opposes age‐associated cortical bone loss.

Although Piezo1 knockout mice initially showed a thinner cortex at young age, they still experienced age‐related cortical bone loss. This suggests that the underlying driving force for age‐associated cortical bone loss is not dependent on Piezo1 signaling. However, our study also found that Piezo1 knockout mice experience more cortical bone loss with age than control mice, indicating that Piezo1 plays an important role in age‐associated cortical bone loss as a defensive factor. This is consistent with previous studies showing that hindlimb unloading, which reduces mechanical stimulation, causes cortical bone loss in old rats (Perrien et al., 2007). Together, these studies suggest that bones in old age still experience some degree of mechanical loading, which can protect them from age‐related cortical bone loss. Additionally, our study found that Piezo1 expression decreases with age in bone, which may contribute to the decreased skeletal response to loading seen with age. Our data provide evidence supporting the idea that reduced mechanical stimulation due to decreased physical activity and/or decreased response to loading contributes to age‐related cortical bone loss.

The protective effects of mechanical loading on cortical bone may be attributed to increased bone formation, decreased bone resorption, or both. Studies in young and adult mice have found that mechanical loading increases bone formation (Li et al., 2019; Lynch et al., 2011), but this ability is greatly reduced in older mice (Holguin et al., 2014; Meakin et al., 2015). Our previous research conducted using RANKL conditional knockout mice has shown that age‐related cortical bone loss is primarily due to endocortical bone resorption rather than decreased bone formation (Kim et al., 2020). Therefore, it is possible that mechanical loading alleviates age‐related cortical bone loss by regulating bone resorption. Our findings are in line with this idea, as we observed that *Piezo1* conditional knockout mice experienced more cortical bone loss with age than control mice due to a more profound increase in endocortical bone resorption. These data suggest that the primary role of Piezo1 signaling in older bones is to harness osteoclast formation in the endosteum and prevent cortical thinning. Whether the effect of Piezo1 signaling on old bone is dependent on mechanical stimulation needs further investigation.

Despite cortical bone loss with age, there is continuous expansion of periosteal bone throughout life (Jepsen & Andarawis‐Puri, 2012). The factors that affect periosteal expansion are not fully understood, but it is thought to be related to changes in hormones, bone remodeling, and mechanical stimulation (Allen et al., 2004; Orwoll, 2003; Parfitt, 2002). In previous studies, we found that Piezo1 deletion in osteoblast lineage cells greatly reduced periosteal bone formation and cortical bone accrual in young growing mice (Li et al., 2019). However, aged Piezo1 knockout mice showed increased endocortical bone resorption but normal periosteal bone formation compared to wildtype mice. These data indicated that Piezo1‐mediated mechanical signaling controls periosteal bone formation in young but not old mice and that periosteal bone formation is not related to bone resorption. Consistent with this, we found in our previous studies that periosteal expansion was not affected in aged RANKL conditional knockout mice in which bone remodeling was greatly suppressed (Kim et al., 2020). More importantly, the bones of RANKL knockout mice are mechanically underloaded since they have much higher bone mass with similar body weight comparing to wildtype mice. Together, these findings suggest that mechanical stimulation plays a minimal role in periosteal bone formation in old mice.

Our study revealed that Piezo1 plays a role in regulating osteoclast formation through its ability to stimulate OPG expression. Elevated production of RANKL by senescent osteocytes has been shown to be a key mechanism responsible for increased bone resorption and age‐related cortical bone loss (Kim et al., 2020). OPG, acting as a decoy receptor for RANKL, helps counteract this effect, but its expression decreases with age (Piemontese et al., 2017), which could contribute to age‐associated bone resorption. We observed a correlation between decreased Piezo1 expression, reduced OPG expression, increased osteoclast formation on the endocortical surface, and further cortical bone deterioration of aged bones. Our results highlight the intricate interplay between age, mechanical loading, and bone resorption, and suggest that lack of mechanical stimulation in aged mice could exacerbate the decline in OPG expression, leading to greater bone resorption and cortical bone degradation.

In contrast to cortical bone, we did not observe a significant cancellous bone loss associated with aging in *Piezo1* conditional knockout mice. One explanation could be that the cancellous bone mass in mice lacking *Piezo1* is already extremely low in young age so that there is not much bone to lose with age in these mice. The second reason could be that Piezo1 signaling primarily affects bone resorption in old age which is related to cortical bone loss while cancellous bone loss is mainly associated with decrease in bone formation. Our results are in line with previous evidence that different aging mechanisms underlie the loss of cortical versus cancellous bone and the difference may be related to the ways the two compartments respond to mechanical loading.

In summary, our findings suggest that mechanical loading primarily promotes bone formation in cortical bone in young growing mice, resulting in increased bone mass and size. However, in old mice, mechanical loading primarily inhibits bone resorption and prevents cortical thinning and porosity. Consistent with this, the role of Piezo1 shifts from promoting bone formation to suppressing bone resorption as mice age. These findings shed light on the intricate mechanisms underlying age‐related bone loss and emphasize the importance of mechanical stimuli in preserving skeletal integrity during aging. This knowledge could inform strategies for preventing age‐related bone disorders.

## 4 MATERIALS AND METHODS

### 4.1 Mice

The generation of mice harboring *Piezo1* conditional allele, termed Piezo1^f/f^ mice, was described previously (Cahalan et al., 2015). The 8 kb Dmp1‐Cre transgenic mice were described previously (Bivi et al., 2012). To generate Dmp1‐Cre;Piezo1^f/f^ mice and littermates, we mated Piezo1^f/f^ mice (crossed into C57BL/6J for more than 10 generations) and Dmp1‐Cre mice (crossed into C57BL/6J for more than 10 generations). C57BL/6J mice were purchased from the Jackson laboratory. An 8 N axial load was applied to left tibia of 4‐month‐old female C57BL/6J mice at the tibial midshaft using an Electroforce TA 5500 (TA Instruments, New Castle, DE). Each mouse was loaded with one bout (less than 10 min) of compressive loading applied in 1200 cycles with 4‐Hz triangle waveform and 0.1 s rest time between each cycle, a protocol shown to be anabolic previously (Li et al., 2019). The unloaded contralateral tibiae were used as controls. Mice were then returned to housing cage and were harvested 4 h later for gene expression analysis. We housed all mice in the animal facility of the University of Arkansas for Medical Sciences. The Institutional Animal Care and Use Committee of the University of Arkansas for Medical Sciences approved protocols involving these mice.

### 4.2 Human bone samples

De‐identified cortical bone samples were collected from human femoral neck removed during hip replacement surgeries. The exclusion criteria are pregnancy, hormonal treatment for gender dysphoria, history of malabsorptive syndromes (e.g., Crohn's colitis, ulcerative colitis, cystic fibrosis, and history of Roux‐en‐Y gastric bypass surgery), solid organ or bone marrow transplants, or chronic kidney disease stage 4 and 5. The samples were carefully selected to avoid abnormal tissue. Total 11 male and 11 female patients were included. The young group had 4 males and 5 females. The aged group had 7 males and 6 females. All participates signed the informed consent and HIPAA Research Authorization forms. The mean age of the young group (*n* = 9) is 47.1 ± 7.01 years old, and the aged group (*n* = 13) is 73.8 ± 3.2 years old. This study is IRB‐approved.

### 4.3 Cell cultures

HEK 293 T cells were authenticated by ATCC. MLO‐Y4 cells were created and authenticated in Dr. Lynda Bonewald's laboratory (Kato et al., 1997). MLO‐Y4 cells were cultured in α‐MEM supplemented with 5% FBS, 5% BCS, and 1% penicillin/streptomycin/glutamine. Bone marrow cells were isolated from 6‐month‐old or 24‐month‐old C57BL/6J mice. For Yoda1 treatment, cells were cultured in the presence of 10 μM Yoda1 (Sigma) or DMSO for 2 h. For calcium free medium, cells were serum starved for 1 h and then treated with Veh or 10 μM Yoda1 for 2 h. For treatment of inhibitors of different pathways, cells were pretreated with inhibitors in αMEM for 2 h and then treated with Veh or Yoda1 in the presence of inhibitors for 2 h. The final concentration for BAPTA is 5 μM, for CMZ is 1.5 μM, for PP242 (mTOR inhibitor) is 2.5 μM, for SB203590 (p38/MAPK inhibitor) is 10 μM, for PD98059 (MEK/ERK inhibitor) is 50 μM, and for L‐NAME (eNOS inhibitor) is 200 μM. Immediately after the treatments, we isolated RNA from cells using RNeasy mini kit (Qiagen, German) for quantitative PCR. Three replicates were included for each experiment and were repeated at least two times. To silence *Piezo1*, we used the *Piezo1* shRNA expression plasmid generated previously (Li et al., 2019). For virus production, HEK293T cells were cultured in a 6‐well culture plate and co‐transfected with a total 3 μg of lentiviral shRNA vector, pMD2G (Addgene plasmid #12259, a gift from Didier Trono), and psPAX2 (Addgene plasmid # 12260, a gift from Didier Trono) at the ratio of 2:0.9:0.4 using TransIT‐LT1 transfection reagent (Mirus, Madison, WI). Culture media was changed 12 h after transfection, and viral supernatants were collected 48 h after media change. Viral supernatants were filtered through a 0.45 μm filter and used immediately to transduce cells cultured in a 10 cm dish. Cells were then subjected to selection with puromycin (25 μg/mL) for 5 days before treatment.

### 4.4 Osteoclast culture

To demonstrate the ability of *Piezo1* knockdown MLO‐Y4 cells to support osteoclast formation, we utilized osteoclast coculture system which has been used to generate osteoclasts in vitro previously (Xiong et al., 2018). Bone marrow cells were isolated from the femurs and tibias of 3‐month‐old male C57BL/6J mice and were cultured in a petri dish in αMEM supplemented with 15% FBS and 100 ng/mL M‐CSF for 6 days to generate macrophages. Bone marrow macrophages were then co‐cultured with MLO‐Y4 cells expressing shRNA for luciferase or Piezo1 in αMEM supplemented with 10% FBS and 10 ng/mL M‐CSF for 7 days to generate osteoclasts. The culture medium was changed every 2 days. At day seven, the cells were fixed in 10% buffered formalin and osteoclasts were visualized by TRAP staining or harvested for RNA isolation and osteoclastic genes were measured. For Yoda1 treatment, bone marrow macrophages were co‐cultured with MLO‐Y4 cells in αMEM supplemented with 10% FBS and 10 ng/mL M‐CSF and in the presence of 2 μM Yoda1 for 7 days. Cells were harvested for RNA isolation and osteoclastic genes were measured.

### 4.5 Femoral organ culture

Female mice at 4 months of age were euthanized in a CO_2_ chamber. Femurs were dissected, and both ends were removed in a culture hood. Bone marrow was then flushed out using PBS, and the periosteal surface was scraped to remove periosteal cells. Femoral shafts were then cultured in a 12‐well‐plate with 1 mL of α‐MEM supplemented with 10% FBS and 1% PSG for 24 h. We then treated femur shafts with 2 μM Yoda1 (Sigma) or DMSO for 3 days. Femur shafts were then collected for RNA isolation and qPCR analysis.

### 4.6 Skeletal analysis

Tibial X‐rays were obtained using an UltraFocus X‐ray machine (Faxitron Bioptics, Tucson, Arizona). Three‐dimensional bone volume and architecture of L4 vertebra and femur were measured by μCT (model μCT40, Scanco Medical). The femur and vertebrae (L4) were cleaned of soft tissues and fixed in 10% Millonig's formalin for 24 h. Bone was then gradually dehydrated into 100% ethanol. Bone samples were loaded into a 12.3 mm diameter scanning tube and images acquired in the μCT40. The scans were integrated into 3D voxel images (1024 × 1024 pixel matrices for each individual planar stack), and a Gaussian filter (sigma = 0.8, support = 1) was used to reduce signal noise. Scanco Eval Program v.6.0 was used for measuring bone volume. A threshold of 220 mg/cm^3^ was applied to all scans at medium resolution (*E* = 55 kVp, *I* = 145 μA, integration time = 200 ms) for trabecular bone measurements. The cortical bone and the primary spongiosa were manually excluded from the analysis. Trabecular bone measurements in the vertebra were determined using 100 slices (1.2 mm) of the anterior (ventral) vertebral body immediately inferior (caudal) to the superior (cranial) growth plate. All trabecular measurements were made by drawing contours every 10–20 slices and voxel counting was used for bone volume per tissue volume and sphere filling distance transformation indices, without pre‐assumptions about the bone shape as a rod or plate for trabecular microarchitecture. Femoral cortical thickness, periosteal circumference, and endocortical circumference were measured at the mid‐diaphysis. Cortical porosity was measured at the distal half of femur. Cortical analyses were measured at a threshold of 260 mg/cm^3^. Calibration and quality control were performed weekly using five density standards, and spatial resolution was verified monthly using a tungsten wire rod. We based beam‐hardening correction on the calibration records. Corrections for 200 mg hydroxyapatite were made for all energies.

### 4.7 Histology

Murine femurs were fixed for 24 h in 10% Millonig's formalin, dehydrated into 100% ethanol, and embedded in methyl methacrylate, and then, 5 μm longitudinal sections were obtained. After removal of plastic and rehydration, we stained sections for TRAP activity and counterstained with T‐blue. Quantitative histomorphometry was performed to determine osteoclast number using Osteomeasure system (OsteoMetrics) interfaced to an Zeiss Axio Imager M2 (Carl Zeiss).

### 4.8 RNAscope

For RNAscope, murine femurs and bone samples from human femoral neck were fixed for 24 h in 10% Millonig's formalin, decalcified in 14% EDTA, dehydrated into 100% ethanol, and embedded in paraffin, and then, 7 μm longitudinal sections were obtained. *Piezo1* and *Tnfrsf11b* mRNA were detected in paraffin sections using RNAscope 2.5 HD Assay RED (Advanced Cell Diagnostics) according to manufacturer's instruction. Specifically, bone sections were deparaffinized and treated with 3% H_2_O_2_ at room temperature for 10 min to inactivate endogenous peroxidase. Sections were then treated with Custom Pretreatment Reagent (Cat# 300040, Advanced Cell Diagnostics) for 30 min to permeabilize the tissue. After pretreatment, probe hybridization was performed using probes for *Piezo1* (Cat# 500511 for mouse and Cat# 485101 for human, Advanced Cell Diagnostics, CA) and *Tnfrsf11b* (Cat# 488961, Advanced Cell Diagnostics), respectively, for 2 h at 40°C in HybEZ II oven (Advanced Cell Diagnostics). Sections were then incubated sequentially with AMP1, AMP2, AMP3, AMP4, AMP5, and AMP6 to amplify and label hybridization signals. Chromogenic substrate was then prepared by mixing Fast RED‐A and ‐B at 60:1 ratio, and sections were incubated with substrate for 10 min at room temperature to detect hybridization signals. After staining, sections were counterstained with 25% hematoxylin and blued using 0.02% ammonia water. Sections were then completely dried at 60°C for 15 min and mounted with EcoMount mounting medium for imaging using Zeiss Axio Imager M2 microscope. The signals were quantified using the ImageJ (NIH, MA).

### 4.9 Quantitative PCR

Organs and whole bones were harvested from animals, removed of soft tissues, and stored immediately in liquid nitrogen. We prepared osteocyte‐enriched cortical bone by removing the ends of femurs or tibias and then flushing the bone marrow with PBS. We then scraped the bone surface with a scalpel and froze them in liquid nitrogen for RNA isolation. We isolated total RNA using TRIzol (Life technologies), according to the manufacturer's instructions and prepared cDNA using High Capacity first strand cDNA synthesis kit (Life Technologies). We performed quantitative RT‐PCR using the following Taqman assays from Applied Biosystems: *Piezo1* (Mm01241549_m1); *Tnfsf11* Mm00441906_m1; *Tnfrsf11b* (Mm00435452_m1); and ribosomal protein S2 (*Mrps2*) (for, 5′‐CCCAGGATGGCGACGAT‐3′, rev, 5′‐CCGAATGCTGTAATGGCGTAT‐3′, probe, 5′‐FAM‐TCCAGAGCAGGATCC‐NFQ‐3′); human *Piezo1* (Hs00207230_m1); human *Mrps2* (Hs00211334_m1); and human *Actb* (Hs03023943_g1). We calculated relative mRNA amounts using the ^∆^Ct method (Livak & Schmittgen, 2001).

### 4.10 Quantification and statistical analysis

GraphPad Prism 9 software (GraphPad, San Diego) was used for statistical analysis. Two‐way analysis of variance (ANOVA) or Student's *t* test were used to detect statistically significant treatment effects, after determining that the data were normally distributed and exhibited equivalent variances. All *t* tests were two‐sided. *p*‐values less than 0.05 were considered as significant. Error bars in all figures represent SD.

## AUTHOR CONTRIBUTIONS

Conceptualization: X.L. and J.X.; Methodology: X.L., C.Z., H.B., J.S., B.S., S.M., L.B., E.A., and J.X.; Investigation: X.L., C.Z., H.B., and J.X.; Formal Analysis: X.L., and J.X.; Writing, original draft: J.X.; Writing, reviewing, and editing: all authors.

## CONFLICT OF INTEREST STATEMENT

The authors declare no conflict of interest.

## Supporting information


Figure S1

## Data Availability

Data sharing is not applicable to this article as no datasets were generated or analyzed during the current study.
