# Supplementary material for: Piezo1 opposes age‐associated cortical bone loss
Source: Aging Cell. 2023 May 5;22(6):e13846. doi: 10.1111/acel.13846 (PMC10265162; doi:10.1111/acel.13846)
Supplement: Supplementary file 1 — Figure S1 [file ACEL-22-e13846-s001.pdf]

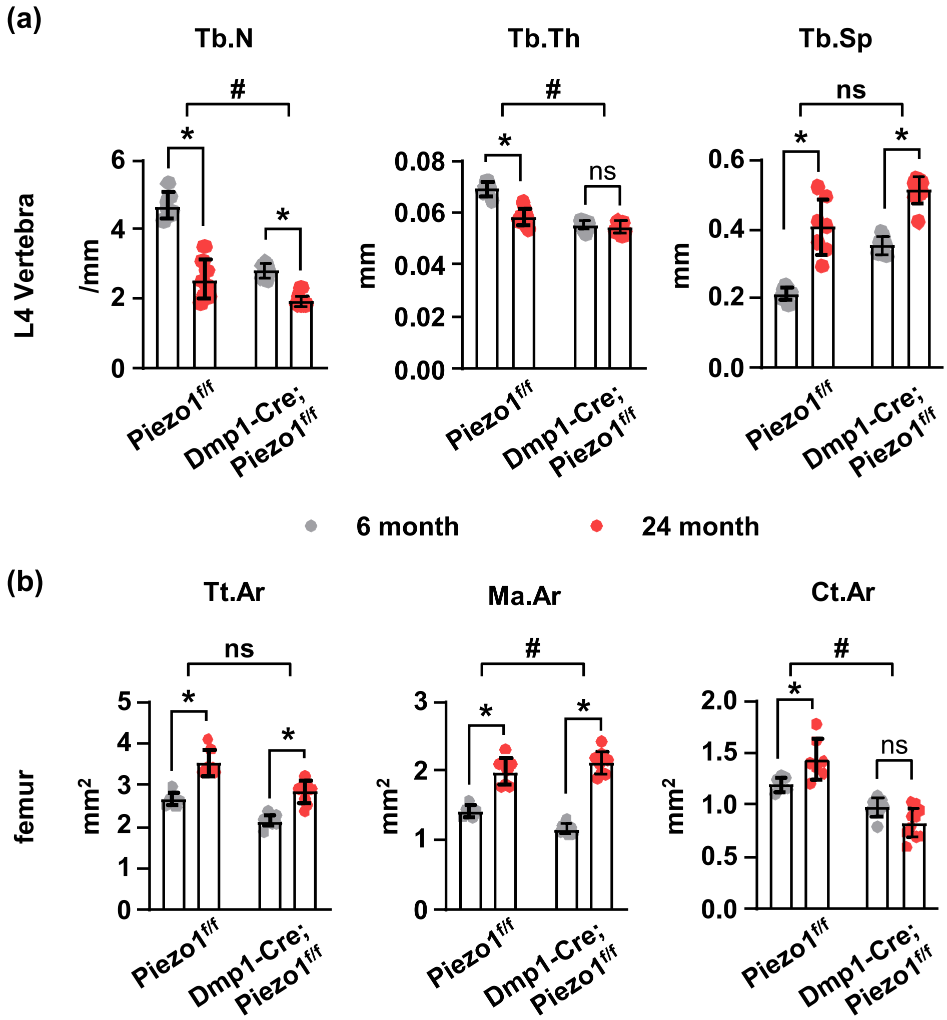

Supplementary Figure 1. MicroCT analysis of cancellous bone in L4 vertebra and cortical bone in femoral diaphysis. (a) Trabecular number (Tb.N), trabecular thickness (Tb.Th), and trabecular separation (Tb.Sp) of vertebral cancellous bone measured in the 4th lumbar vertebra of 6- and 24-month-old female $Piezo1^{f/f}$ (n =8, 10) and Dmp1-Cre;$Piezo1^{f/f}$ (n = 8, 12) mice. (b) Total cross-sectional area (Tt.Ar), medullary area (Ma.Ar), and cortical area (Ct.Ar) measured in the femoral diaphysis of 6- and 24-month-old female $Piezo1^{f/f}$ (n =8, 10) and Dmp1-Cre;$Piezo1^{f/f}$ (n =8, 12) mice. *$p < 0.05$ with the comparisons indicated by the brackets using 2-way ANOVA. #, $p < 0.05$ for interaction using 2-way ANOVA. ns, nonsignificant.
